# The Financial Burden of Prescribed Medications in COPD

**DOI:** 10.1016/j.chpulm.2026.100277

**Published:** 2026-06-11

**Authors:** Jeenat Mehareen, Mohsen Sadatsafavi, Phalgun Joshi, Chris Carlsten, Joseph Emil Amegadzie

**Affiliations:** a Respiratory Evaluation Sciences Program, Faculty of Pharmaceutical Sciences, University of British Columbia, Vancouver, BC, Canada; b Collaboration for Outcomes Research and Evaluation, Faculty of Pharmaceutical Sciences, University of British Columbia, Vancouver, BC, Canada; c The Legacy for Airway Health and Centre for Lung Health, Vancouver Coastal Health Research Institute, Vancouver, BC, Canada; d Division of Respiratory Medicine, University of British Columbia, Vancouver, BC, Canada; e British Columbia Centre for Disease Control, Vancouver, BC, Canada

**Keywords:** COPD, excess costs, financial burden, inhaler, medications, private financing

## Abstract

**Background:**

Medication costs are an important component of chronic disease burden. Among individuals with COPD, medication costs have increased consistently over time.

**Research Question:**

What excess medication costs are observed among individuals with COPD compared with those without COPD, and what share of these costs is financed privately through out-of-pocket or private insurance?

**Study Design and Methods:**

Using administrative health databases from British Columbia, Canada (2001-2020), we created a retrospective cohort of patients with physician-diagnosed COPD matched to individuals without COPD on demographic and socioeconomic characteristics. We estimated excess direct medication costs as the adjusted difference in all-cause medication costs between individuals with and without COPD, using generalized linear models. Primary outcomes included total excess medication costs, privately financed excess medication costs, defined as prescription medication costs minus PharmaCare contributions, and inhaler costs among patients with COPD. Trends were assessed using the average annual percentage change. Costs were reported in 2020 Canadian dollars.

**Results:**

The final sample consisted of 207,502 individuals with COPD and 403,941 individuals without COPD (mean baseline age, 68.9 and 68.4 years; 52.4% and 51.3% male, respectively). Average overall excess medication costs were $1,336 (95% CI, 1,321-1,352) per patient-year (PY), of which 40% ($528/PY; 95% CI, $521-$535) were privately financed, and increased more rapidly than PharmaCare-financed spending (average annual percentage change, 8.3% vs 3.8%). Patients with COPD bore one-half of the inhaler costs ($179/PY of $352/PY).

**Interpretation:**

Our results show that individuals with COPD have higher all-cause medication costs and a growing private cost burden compared with those without COPD.

**Take-Home Points**

**Study Question:** Among individuals with COPD, what are the excess medication costs and how much is paid privately compared with matched individuals without COPD over 20 years?

**Results:** Individuals with COPD incurred excess medication costs of $1,336 per patient-year, of which 40% ($528) were privately financed, and increased more rapidly than PharmaCare (8.3% vs 3.8%). One-half of inhaler costs were paid out-of-pocket or via private coverage.

**Interpretation:** Our results indicate that COPD places a substantial medication cost burden on patients, and it is increasing.

COPD is a globally prevalent respiratory condition, contributing significantly to morbidity and mortality.^1^ Pharmacotherapy, including inhaled medications, plays an important role in contemporary COPD management.^2^, ^3^, ^4^ However, medication costs, in particular inhaler costs, have risen substantially over the past 2 decades.^5^ It is estimated that the annual medical costs for COPD among adults ≥ 45 years of age are US $24.0 billion, with US $11.9 billion attributed to medication costs (in 2018 US dollars).^6^ The investigators reported a 72% increase in the average annual medical costs of COPD between 2000 and 2018, accompanied by an 8-fold rise in prescription drug costs over the same period.^6^ Consistent with this trend, higher medication expenses have been associated with increased cost-related nonadherence among older adults with airway disease,^7^ and with higher hospitalization rates.^8^^,^^9^

In Canada, legal residents benefit from a provincially administered universal health care system covering hospital and physician services at no cost. However, prescription drug coverage is fragmented, relying on a mix of public and private financing (ie, private insurance, out-of-pocket payments).^10^ Federal and provincial governments set coverage, eligibility, and cost-sharing by age, income, and medical condition. Despite this mixed financing model, national drug expenditures have risen substantially, from CAD $1.7 billion in 2000 to CAD $32.7 billion in 2020 (adjusted to 2020 currency values) and are projected to increase by 3.8% annually.^11^ At the patient level, costs have also escalated. For example, average annual per-patient costs of inhaled medications nearly doubled between 1997 and 2015 in COPD.^12^

COPD is a progressive condition that remains an important contributor to morbidity among adults. Although prevalence among younger adults has declined,^13^^,^^14^ the burden remains considerable among individuals with severe disease, particularly those unable to work and requiring multiple medications for comorbidities.^15^ The population-based administrative data in British Columbia, Canada, provide a powerful source to quantify the burden of medication costs. As such, the purpose of this study was to quantify the excess overall and privately financed (through out-of-pocket or private insurance coverage) all-cause medication costs among individuals with COPD compared with matched individuals without COPD, and inhaler costs among individuals with COPD.

## Study Design and Methods

### Data Source

We used anonymized data from January 1997 to December 2022 from the provincial health administrative databases of British Columbia.^16^ As of 2021, British Columbia (the third largest province in Canada with around 5.0 million residents) manages centralized databases to record health care usage data for all residents, irrespective of age, immigration status, or third-party insurance coverage. Access to data provided by the Data Stewards is subject to approval but can be requested for research projects through the Data Stewards or their designated service providers. The following data sets were used in this study: Consolidation File (includes demographics, registry, and census geodata), PharmaNet, Vital Events and Statistics - Births, and Vital Events and Statistics - Deaths. You can find further information regarding these data sets by visiting the PopData project webpage.^17^ All inferences, opinions, and conclusions drawn in this publication are those of the authors, and do not reflect the opinions or policies of the Data Steward(s). Ethical approval was obtained from the University of British Columbia human ethics board (No. H21–070).

### Study Population

We constructed a retrospective matched cohort comprising adults with and without diagnosed COPD. We used a validated criterion to define COPD diagnosis that required at least 1 COPD-related hospitalization (determined using International Classification of Diseases [ICD] ninth revision [ICD-9] or 10th revision [ICD-10] codes) or a minimum of 3 COPD-related outpatient visits within a continuous 24-month time window.^18^ The ICD-9 codes included 491, 492, 493.2x, and 496, whereas the ICD-10 codes included J41, J42, J43, and J44. This case definition has demonstrated a high specificity of 95.4% and a positive predictive value of 81.3% among people living with COPD.^18^ To further enhance the specificity of the COPD case definition and exclude patients with potential asthma, we limited our analysis to individuals ≥ 45 years of age at the time of the first COPD-related record in the data set. For each patient identified in the COPD cohort, we found up to 2 matched individuals from the general non-COPD population (any individual who was not a member of the COPD cohort). Matching was based on biological sex, year of birth (± 5), local health service delivery area, and socioeconomic status. Socioeconomic status was defined using census-derived (measured at the finest geographic level), equivalized household income-based neighborhood quintiles. The purpose of matching was to create a relevant comparison group by important sociodemographic factors. We did not control clinical variables such as the severity of COPD or burden of comorbidities because our study was not aimed at estimating the causal association between COPD and excess costs.

In the administrative health data, disease status before the start of available records is unknown. To distinguish true incident cases from preexisting (prevalent) cases, and following prior literature,^19^^,^^20^ we used the first 4 years of available data as a wash-in period and excluded them from the cost calculation. Similarly, the last 2 years (2021 and 2022) of data were also omitted from the analysis because of the 2-year follow-up requirement for the case definition of COPD (which would result in a partial identification of COPD cases in the last 2 years). As such, the study period was defined as January 1, 2001, to December 31, 2020. Schematic illustration of the study design is provided in e-Figure 1.

The entry date for each patient was determined as the first date of any recorded information in the data set. For patients who met the COPD case definition, we designated the index date as the day after their initial COPD diagnosis (to prevent bias from assured resource utilization on the index date for the COPD cohort, unlike the comparison group). The index date marked the beginning of the follow-up period (time zero). The index date for the individuals in the comparison cohort was selected to match that of their respective patient with COPD. Follow-up continued until the earliest occurrence of any of the following events: death, the end of the study period, the end of registration from the health care system, or the last recorded use of any health care resource.

### Outcome Measures

The primary outcome was the total and privately financed excess all-cause medication costs. Excess costs were defined as the difference in costs between COPD and a comparable non-COPD group. Total medication costs and corresponding PharmaCare (public payer) contributions were obtained from the PharmaNet database. PharmaNet captures all prescriptions dispensed in community and outpatient pharmacies in British Columbia but does not include information on third-party private insurance coverage or patient out-of-pocket payments separately. Therefore, privately financed costs were estimated by subtracting the public PharmaCare contribution from total prescription medication costs.

The secondary outcomes were total and privately financed inhaled medication costs among patients with COPD. These medications were identified by unique identification numbers according to a predetermined list.^21^^,^^22^ Inhaler dispensations were included for any individuals meeting the COPD case definition because prescription records do not specify treatment indication.

### Statistical Analysis

We predetermined the statistical significance level at 0.05. Adjusted excess costs were estimated using generalized linear models with generalized estimating equations to account for clustered data (ie, patient years within COPD and non-COPD matched pairs). We used a normal distribution and an identity link function, which is shown to be robust against skewed cost data when the sample size is large.^23^ Regression analyses were conducted with overall excess costs of COPD per patient-year (PY) as the outcome variable. Independent variables included cohort membership (coded as 1 for the COPD cohort and 0 for the comparison cohort), calendar time, age group at the index date, sex, and local health-delivery area to control for any remaining imbalances after matching. The regression coefficient associated with cohort membership represented the adjusted excess costs of COPD. Separate regression models were applied to analyze total medical costs, PharmaCare, and privately financed plans. We also reported the average annual percentage change (AAPC) to show the trend in excess costs over the 20-year period. It was derived from the year coefficient of a log-transformed model. Costs were grouped by calendar year and adjusted to 2020 Canadian dollars.

### Subgroup and Sensitivity Analyses

We conducted subgroup analyses stratifying patients into younger and older adult age groups (45-64 and ≥ 65 years) and by sex. Sensitivity analyses included refitting models using a gamma distribution with identity link commonly used to account for right-skewed cost data. To evaluate the potential effect of informative censoring from end-of-life expenditures, we repeated analyses after excluding the terminal patient year (year of death) separately within the COPD and comparison cohorts.

All statistical analyses were conducted using SAS version 9.4 (SAS Institute, Cary, NC, USA) and R version 4.2.2 (www.r-project.org).

## Results

The COPD cohort included 207,502 patients, who were matched with 403,941 individuals from the general population without COPD. On the index date, the mean ages ± SD of the COPD and non-COPD cohorts were 68.9 ± 11.6 and 68.4 ± 11.4 years, respectively. In total, 52.4% of the COPD and 51.4% of the non-COPD cohorts were male. The mean follow-up time for the COPD and comparison cohorts was 7.6 ± 4.3 and 7.7 ± 4.5 years, respectively. Among those with COPD, 37% were 45 to 64 years of age, and 25.5% resided in the lowest income quintile neighborhoods. The baseline characteristics of the COPD group closely mirrored those of the matched non-COPD cohort across all variables (Table 1).

**Table 1:** Baseline Characteristics of COPD and Non-COPD Cohorts

Characteristic	COPD (n = 207,502)	Non-COPD (n = 403,941)^a^
Follow-up, y	7.6 [4.3]	7.7 [4.5]
Sex		
Female	47.6	48.7
Male	52.4	51.3
Age, y	68.9 [11.6]	68.4 [11.4]
Age group, y		
45-59	23.4	24.0
60-74	43.3	45.1
≥ 75	33.3	30.9
Socioeconomic status		
Quintile 1	25.5	24.7
Quintile 2	21.1	21.2
Quintile 3	18.9	18.9
Quintile 4	17.5	17.5
Quintile 5	15.3	16.1
Unknown	1.7	1.7
Charlson Comorbidity Index score	4.3 [3.2]	2.3 [2.8]
Comorbidities		
Myocardial infarction	14.1	6.8
Congestive heart failure	25.8	10.4
Peripheral vascular disease	20.5	10.0
Cerebrovascular disease	18.5	11.2
Cancer	32.3	21.9
Dementia	9.4	6.8
Rheumatic disease	19.6	11.6
Peptic ulcer disease	14.3	8.2
Paraplegia or hemiplegia	2.9	1.7
Renal disease	14.6	8.2
Liver disease	14.0	8.5
Diabetes	33.6	26.5
AIDS/HIV	0.8	0.4

Data are presented as mean [SD], %, or as otherwise indicated.

a Charlson Comorbidity Index at the time of COPD diagnosis (without age adjustment) was calculated cumulatively using all available data before the index date.

**Figure 1:**
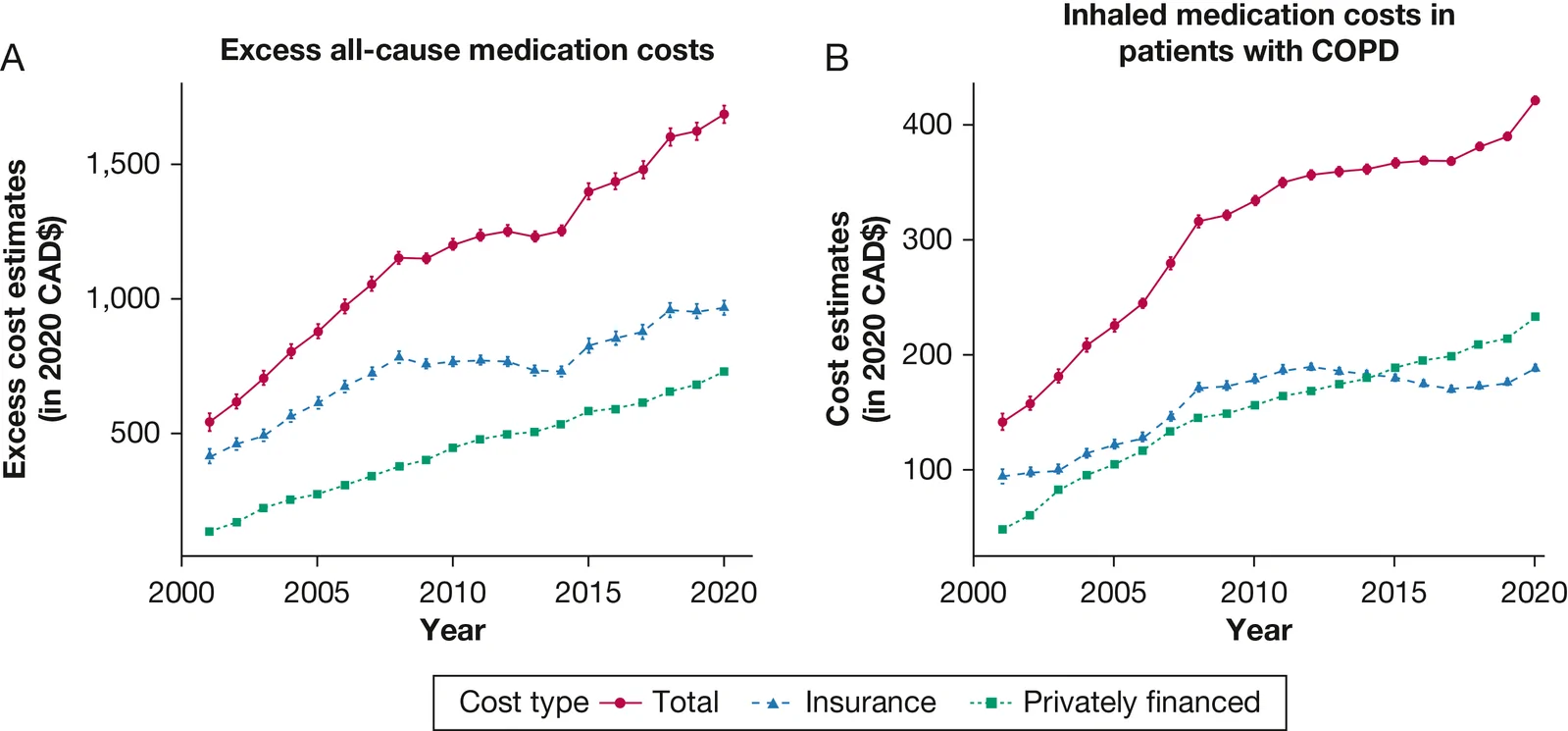
A, B, Trends in adjusted annual excess direct all-cause medication costs (A) and inhaler medication costs (B) in patients with COPD categorized by cost type. Points are estimated costs, with error bars indicating the 95% CI.

Adjusted annual excess all-cause medication costs were $1,336 per PY (95% CI, $1,321-$1,352) (Table 2). Of these, 40% ($528/PY; 95% CI, $521-$535) were privately financed payments. Excess all-cause medication costs increased 5.4% annually (*P* < .01), with privately financed costs rising faster than PharmaCare (AAPC, 8.3% vs 3.8%) (Fig 1A). Privately financed costs for inhaled medications ($179/PY; 95% CI, $177-$180) accounted for 50% of the total inhaler costs ($352/PY; 95% CI, $349-$354) in patients with COPD. The privately financed share increased from 33% in 2001 to 55% in 2020, surpassing PharmaCare costs after 2014 (Fig 1B). This shift corresponds to 7% AAPC, which was significantly higher than the AAPC of 3.5% for PharmaCare costs (*P* < .01).

**Table 2:** Adjusted Excess All-Cause Medication Costs and COPD-Related Inhaler Medication Costs Per Patient-Year in Total and by Cost Category

Characteristic	Excess All-Cause Medication Costs (95% CI)^a^^,^^b^			Inhaler Medication Cost (95% CI)^a^^,^^b^		
	Total	PharmaCare	Privately Financed	Total	PharmaCare	Privately Financed
Year						
2001-2020	1,336 (1,321-1,352)	808 (796-822)	528 (521-535)	352 (349-354)	173 (171-175)	179 (177-180)
2001	535 (505-567)	408 (381-436)	127 (117-138)	141 (134-148)	94 (88-99)	47 (44-51)
2020	1,686 (1,653-1,719)	962 (936-988)	724 (708-740)	421 (417-442)	188 (185-190)	233 (230-235)
AAPC^c^ %, 2001-2020	5.4 (4.5-6.2)	3.8 (3.0-5)	8.3 (7.1-9.5)	5.1 (4.1-6.2)	3.5 (2.4-4.6)	7.0 (5.7-8.4)
By sex						
Female	1,369 (1,347-1,391)	836 (818-854)	533 (524-543)	365 (361-369)	186 (184-189)	179 (176-181)
Male	1,306 (1,283-1,328)	784 (765-803)	522 (513-532)	338 (334-342)	160 (160-162)	178 (176-181)
Age group, y						
45-64	1,652 (1,621- 1,681)	1,048 (1,022-1,073)	604 (592-616)	363 (359-367)	170 (167-173)	193 (190-196)
≥ 65	1,098 (1,082-1,114)	628 (616-640)	470 (462-478)	340 (337-343)	176 (174-179)	164 (162-166)

AAPC = annual average percentage change.

a All costs (per patient-year) represent annual (per patient-year) values and are adjusted to 2020 Canadian dollars.

b Estimated for the study period (significant at *P* < .01).

c AAPC was calculated using a log-linear regression model.

Excess costs were higher among female individuals ($1,369/PY; 95% CI, 1,347-1,391) than male individuals ($1,306/PY; 95% CI, 1,283-1,328). Also, younger adults (45-64 years of age) had greater excess costs than older adults ($1,652/PY; 95% CI, 1,621-1,681 vs $1,098/PY; 95% CI, 1,082-1,114). However, privately financed costs accounted for a larger share of total costs among older adults than younger adults (43% vs 37%). A similar pattern was observed for inhaler costs in COPD. In contrast with all-cause medications, privately financed inhaler costs were higher among adults 45 to 64 years of age (Table 2).

For all-cause excess medication costs, male individuals exceeded female individuals after 2016, but the difference in costs diminished by 2020. Privately financed costs were similar between sexes for both excess all-cause medication and inhaler-related costs (e-Fig 2).

**Figure 2:**
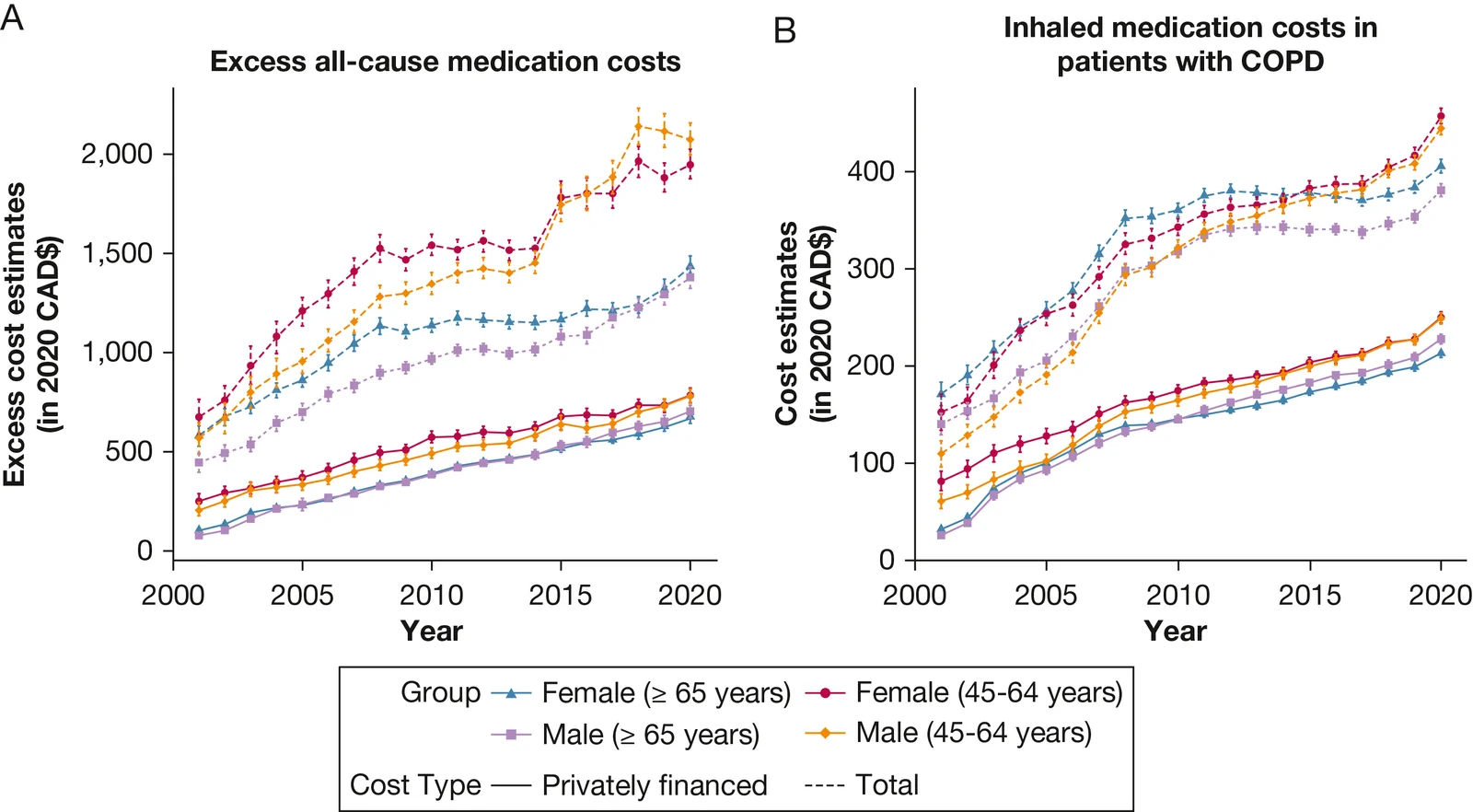
A, B, Trends in adjusted annual excess direct all-cause medication costs (A) and inhaler medication costs (B) in patients with COPD categorized by sex, age, and cost type. Points are estimated costs, with error bars indicating the 95% CI. The dashed lines represent the total medication costs, and the solid lines represent the privately financed medication costs.

In both sexes, overall excess medication costs were higher in younger adults compared with older adults (female: $1,660/PY [45-64 years of age] vs $1,158/PY [≥ 65 years of age]; male: $1,643/PY [45-64 years of age] vs $1,038/PY [≥ 65 years of age]). Similar trends were observed for inhaler costs in patients with COPD, with younger adults incurring higher costs from 2015 onward (Fig 2).

### Sensitivity Analysis

When we excluded the terminal year of life among individuals who died in both the COPD and non-COPD cohorts, the results remained similar to the main analysis. Excess all-cause medication costs were $1,380/PY (95% CI, 1,364-1,397). Similarly, analyses assuming a gamma distribution yielded comparable estimates to the primary analysis (see e-Table 1 for full results).

## Discussion

In this large, population-based matched cohort from British Columbia, Canada (2001-2020), individuals with COPD experienced substantial and rising excess medication costs relative to matched comparators without COPD. Privately financed payments, through out-of-pocket spending or private insurance coverage, accounted for 40% of excess costs ($528/PY; 95% CI, $521-$535 of $1,336/PY; 95% CI, $1,321-$1,352) and increased faster than total costs (8.3% vs 5.4% annually). Private payments also represented one-half of inhaler costs and increased from 33% to 55% during follow-up. Higher costs among females and younger adults point to widening disparities in financial burden.

Previous studies have reported that rising respiratory care costs reflect trends such as higher prices and greater treatment intensity, particularly for pharmacotherapies.^5^ Studies from Canada, the US, and Korea similarly report growth in the medication component of COPD care relative to hospitalization costs.^3^^,^^19^^,^^24^, ^25^, ^26^ The shift toward newer combination inhalers, many of which lack generic substitutes, likely contributes to escalating unit costs. For example, between 2012 and 2018, Medicaid spent $26.2 billion (2018 US dollars) on inhalers, with inhaled corticosteroid containing inhalers contributing to a higher burden.^27^ In addition, British Columbia’s Fair PharmaCare program is structured around income-tested deductibles and 30% coinsurance until reaching a family maximum.^28^ This can lead to sizeable patient-shared expenses for those who do not meet thresholds early or whose income places them just above subsidy cut points. Evidence from the US^29^ and Germany^30^ similarly shows that rising patient cost-sharing reduces access to essential medications.

Although insurer-paid and out-of-pocket components could not be separated, the increasing share of private financing is concerning. Higher private contributions may reflect rising deductibles or copayments, increasing financial burden even among insured individuals.^31^ This pattern was most evident among younger adults (45-64 years of age), who represented 37% of the COPD cohort and had higher excess inhaler costs than older adults. Differences in coverage likely contribute to this finding because younger adults rely more on private or employment-linked insurance, whereas older adults more often access public programs such as British Columbia PharmaCare. This concern is especially relevant for people living with COPD who require ongoing pharmacotherapy. Previous studies show that younger individuals, those without drug insurance, and those with lower income are more likely to experience medication affordability barriers and reduced adherence. Despite income-based protections within PharmaCare,^28^ private medication spending among individuals with COPD grew faster than public spending in our analysis. Policies such as income-adjusted copayments, catastrophic caps, or first-dollar coverage for essential therapies could reduce these disparities.^32^

These findings also have important implications for current policy developments in Canada. The federal Pharmacare Act includes initial universal coverage for contraception and diabetes medications and mandates the creation of an essential medicines list, a bulk purchasing program, and appropriate-use strategies.^33^ The essential medicines list process provides a concrete opportunity to consider medication use among individuals with COPD. This includes both inhaled therapies and medications used to manage common comorbid conditions, particularly given their central role in COPD management, and the increasing share of privately financed medication costs observed in the present study. Internationally, many Organization for Economic Co-operation and Development countries use cost-sharing caps or progressive reimbursement thresholds (eg, Denmark’s increasing reimbursement rates with higher annual spending)^34^ to balance affordability and sustainability while protecting adherence to long-term therapies. Prioritizing cost-effective medications and reducing copayments may help support adherence and disease control across conditions treated in individuals with COPD, particularly in the context of rising private medication costs. Clinicians and health systems can prioritize cost-conscious prescribing, such as selecting the lowest-cost clinically equivalent inhaler when appropriate, considering generics where available, discussing expected pharmacy costs with patients, and implementing structured medication reviews and adherence supports (eg, synchronizing refills).^35^

Our findings offer insight into medication cost patterns in a large retrospective cohort with extended follow-up. However, the study has limitations. First, we were unable to disaggregate private spending into private insurance vs out-of-pocket within the PharmaNet data. Second, because excess costs were measured using all-cause prescription expenditures, we did not distinguish medications related to COPD from those prescribed for smoking-related comorbid conditions (eg, cardiovascular disease, cerebrovascular disease, lung cancer). These comorbidities are more prevalent among individuals with COPD and are likely to contribute to the observed excess medication costs. Third, over-the-counter and inpatient medications were not captured, likely underestimating total medication burden. Fourth, the data set excludes individuals covered by First Nations and federal assurance plans. Finally, cost estimates are specific to British Columbia’s pricing and drug benefit structure and may not generalize to jurisdictions with different policy environments.

## Interpretation

Our study shows that individuals with COPD have higher all-cause medication costs than matched comparators without COPD, and the increasing share of these costs is financed through out-of-pocket spending or private insurance coverage. This trend is likely to intensify over time. Proactive action is needed to address the financial challenges faced by people living with COPD and to support long-term outcomes.

## Funding/Support

This study was funded by Legacy for Airway Health and the Canadian Institutes of Health Research with a Team Grant to the University of British Columbia [Grant PHT 178432].

## Financial/Nonfinancial Disclosures

The authors have reported to *CHEST Pulmonary* the following: M. S. reports a relationship with AstraZeneca Canada Inc that includes consulting or advisory, speaking and lecture fees, and travel reimbursement. None declared (J. M., P. J., C. C., J. E. A.).

## Supplementary Data

e-Online Data
