# Supplementary material for: The Financial Burden of Prescribed Medications in COPD
Source: CHEST Pulm. 2026 Jun 11;4(3):100277. doi: 10.1016/j.chpulm.2026.100277 (PMC13543011; doi:10.1016/j.chpulm.2026.100277)
Supplement: Supplementary file 1 [file mmc1.docx]

**The Financial Burden of Prescribed Medications in Chronic Obstructive Pulmonary Disease**

Jeenat Mehareen, MA^1,2^, Mohsen Sadatsafavi, MD, PhD^1,2^, Phalgun Joshi, PhD^3^, Chris Carlsten, MD MPH^3,4^, Joseph Emil Amegadzie, MSc, PhD^1,2,5^

**Supplementary Material**

| Year | Excess All-cause Medication Costs^ab^  CAD$ (95% CI) | | | Inhaler Medication Costs^ab^  CAD$ (95% CI) | | |
| --- | --- | --- | --- | --- | --- | --- |
|  | Total | PharmaCare | Privately Financed | Total | PharmaCare | Privately Financed |
| Excluding the terminal patient-year (i.e., the year of death) | | | | | | |
| 2001-2020 | 1380  (1364,1397) | 829  (816, 843) | 551  (544, 558) | 358  (355, 361) | 175  (173-177) | 183  (182, 185) |
| Assuming a gamma distribution | | | | | | |
| 2001-2020 | 1360  (1341, 1374) | 828  (815, 842) | 532  (525, 538) | 353  (350, 355) | 174  (172,176) | 179  (178, 181) |
| a: All costs (per patient-year) represent annual (per patient-year) values and are adjusted to 2020 Canadian dollars  b: Estimated for the study period (significant at *P* < 0.01)  *Abbreviations*: COPD, chronic obstructive pulmonary disease; CI, confidence interval; CAD$, Canadian dollars, AAPC: Annual Average Percentage Change | | | | | | |

**e-Table 1:** Sensitivity analysis of adjusted excess costs medication costs of COPD per patient-year in total and by cost components

**Index Date:**

COPD cohort: 1^st^ COPD Dx

Non-COPD cohort: index date from the matched COPD

**End Date:**

Death, end of registration of 12/31/2020

**Entry Date:** 1997 or later

Cost Assessment Window

2001-2020

Wash-in Period: ≥4 years

# **e-Figure 1:** Schematic presentation of study design

# **e-Figure 2:** Trends in adjusted annual excess direct all-cause medication costs (A) inhaled medication costs (B) in COPD patients categorized by sex and cost type**.**

Points are estimated costs, with error bars indicating the 95% CI. The solid lines represent the total medication costs. The dashed lines represent the privately financed medication costs. Abbreviations: COPD, chronic obstructive pulmonary disease; CI, confidence interval; CAD$, Canadian dollars.
